# Simultaneous measurement of the size and methylation of chromosome 4qA-D4Z4 repeats in facioscapulohumeral muscular dystrophy by long-read sequencing

**DOI:** 10.1186/s12967-022-03743-7

**Published:** 2022-11-08

**Authors:** Yosuke Hiramuki, Yuriko Kure, Yoshihiko Saito, Megumu Ogawa, Keiko Ishikawa, Madoka Mori-Yoshimura, Yasushi Oya, Yuji Takahashi, Dae-Seong Kim, Noriko Arai, Chiaki Mori, Tsuyoshi Matsumura, Tadanori Hamano, Kenichiro Nakamura, Koji Ikezoe, Shinichiro Hayashi, Yuichi Goto, Satoru Noguchi, Ichizo Nishino

**Affiliations:** 1 Department of Neuromuscular Research, National Institute of Neuroscience, National Center of Neurology and Psychiatry, Kodaira, Japan; 2 Medical Genome Center, National Center of Neurology and Psychiatry, Kodaira, Japan; 3 Department of Neurology, National Center Hospital, National Center of Neurology and Psychiatry, Kodaira, Japan; 4 Department of Neurology, Pusan National University Yangsan Hospital, Yangsan, Republic of Korea; 5 Department of Neurology and Cerebrovascular Medicine, Saitama Medical University International Medical Center, Saitama, Japan; 6 Department of Neurology, National Hospital Organization Osaka Toneyama Medical Center, Osaka, Japan; 7 Division of Neurology, Department of Aging and Dementia, Second Department of Internal Medicine, Faculty of Medical Sciences, University of Fukui, Fukui, Japan; 8 Department of Neurology, National Hospital Organization Nishi-Beppu National Hospital, Beppu, Japan; 9 Department of Neurology, Matsuyama Red Cross Hospital, Matsuyama, Japan; 10 Department of Mental Retardation and Birth Defect Research, National Institute of Neuroscience, National Center of Neurology and Psychiatry, Kodaira, Japan

**Keywords:** Facioscapulohumeral muscular dystrophy, D4Z4, DUX4, Nanopore sequencer, CpG methylation, CRISPR/Cas9

## Abstract

**Background:**

Facioscapulohumeral muscular dystrophy (FSHD) is an autosomal dominant muscular disorder characterized by asymmetric muscle wasting and weakness. FSHD can be subdivided into two types: FSHD1, caused by contraction of the D4Z4 repeat on chromosome 4q35, and FSHD2, caused by mild contraction of the D4Z4 repeat plus aberrant hypomethylation mediated by genetic variants in *SMCHD1*, *DNMT3B*, or *LRIF1*. Genetic diagnosis of FSHD is challenging because of the complex procedures required.

**Methods:**

We applied Nanopore CRISPR/Cas9-targeted resequencing for the diagnosis of FSHD by simultaneous detection of D4Z4 repeat length and methylation status at nucleotide level in genetically-confirmed and suspected patients.

**Results:**

We found significant hypomethylation of contracted 4q-D4Z4 repeats in FSHD1, and both 4q- and 10q-D4Z4 repeats in FSHD2. We also found that the hypomethylation in the contracted D4Z4 in FSHD1 is moderately correlated with patient phenotypes.

**Conclusions:**

Our method contributes to the development for the diagnosis of FSHD using Nanopore long-read sequencing. This finding might give insight into the mechanisms by which repeat contraction causes disease pathogenesis.

**Supplementary Information:**

The online version contains supplementary material available at 10.1186/s12967-022-03743-7.

## Background

Facioscapulohumeral muscular dystrophy (FSHD) is an autosomal disease characterized by muscle weakness that initially manifests in the face, shoulder, and upper arms, followed by asymmetric involvement of other muscles [1]. *DUX4* is a causative gene for FSHD and is located within an approximately 3.3 kb repeat sequence, referred to as D4Z4, which comprises 1–100 repeat units (RUs) on the subtelomeric regions of chromosomes 4 and 10. Chromosome 4 has two haplotypes distal of the D4Z4 repeat, 4qA and 4qB, where only the 4qA allele contributes to FSHD development, due to the presence of a polyadenylation signal in the most distal D4Z4 RU [2, 3].

FSHD has two types, FSHD1 and FSHD2, both caused by genetic defects leading to aberrant DUX4 expression in skeletal muscle [4]. FSHD1 is mediated by contraction of the D4Z4 4qA allele to 1–10 RUs [5], while FSHD2 is caused by a combination of milder D4Z4 contraction (8–20 RUs) and genetic variants in *SMCHD1*, *DNMT3B*, or *LRIF1*, which each encode epigenetic modifiers [6–8]. Epigenetic modifiers affect histone modification, DNA methylation, and RNA-based mechanisms, may be involved in mechanisms of various diseases and have important diagnostic potential [9] DNA methylation and histone modification at D4Z4 RUs are altered in FSHD [10–12]. CpG methylation is specifically decreased at the contracted D4Z4 repeat on chromosome 4 in FSHD1, while the D4Z4 repeats on both chromosomes 4 and 10 are hypomethylated in FSHD2 [10, 13, 14]; however, the distribution of methylation throughout the full D4Z4 repeat sequence has not been analyzed.

Southern blotting, bisulfite sequencing, molecular combing, and next-generation sequencing are currently used for genetic diagnosis of FSHD [15], but these diagnostic procedures and interpretation of their results present several difficulties. First, interpretation of hybridization patterns generated by Southern blotting is complicated by the fact that the detecting probe also recognizes an additional locus on chromosome 10q that is almost completely homologous to the target 4q35 locus. Second, two subtelomeric variations distal to D4Z4 have been identified on chromosome 4, referred to as the 4qA and 4qB alleles, and selective identification of contracted 4qA repeats is necessary, as only 4qA is associated with FSHD. Third, analysis of CpG methylation by bisulfite sequencing has been performed across the entire D4Z4 units at both the 4q and 10q loci; however, a focal region of extreme demethylation has been reported [16]. Additionally, several patients with milder D4Z4 contraction and CpG hypomethylation have been identified, making diagnosis difficult.

Here, we applied Nanopore CRISPR/Cas9-targeted resequencing (nCATS) to measure the number of D4Z4 RUs and their methylation status in patients with FSHD. We specifically analyzed D4Z4 RUs derived from 4qA and measured the CpG methylation rate in each RU. D4Z4 RUs from 10q were also analyzed.

## Methods

### Genomic DNA preparation

Peripheral blood lymphocytes (10 ml) were combined with 30 ml EL buffer (155 mM NH_4_Cl, 10 mM KHCO_3_, 1 mM EDTA, pH 7.4) on ice for 15 min, followed by centrifugation (KUBOTA 5930, RS-3012M) (840×*g*, 10 min, room temperature). After a repeat EL buffer wash, pellets were suspended in 3 ml NL buffer (10 mM Tris–HCl, 2 mM EDTA, 400 mM NaCl, pH 8.2), followed by addition of 1% SDS and proteinase K and incubation at 37 °C overnight. DNA lysis solution was added with 1 ml 5 M NaCl, followed by phenol/chloroform extraction and ethanol precipitation. DNA pellets were suspended in TE buffer.

Fibroblasts grown in culture dishes were lysed in 10 mM Tris–HCl, 10 mM EDTA, 150 mM NaCl, pH 8.0 containing 0.5% SDS and proteinase K at 55 °C overnight, followed by phenol/chloroform extraction and ethanol precipitation. DNA pellets were suspended in TE buffer.

### DNA library preparation

DNA libraries were prepared using a ligation sequencing kit (Oxford Nanopore Technologies, SQK-LSK109). To generate Cas9 ribonucleoprotein complexes (RNPs), annealed 1 μM tracrRNA-crRNA pool (CR1/CR2/CR3/CR4) and 0.5 μM HiFi Cas9 were incubated at room temperature (around 23 °C) for 30 min. Genomic DNA (2 μg) was dephosphorylated with Quick Calf Intestinal Phosphatase (NEB, #M0525S) at 37 °C for 10 min, followed by 80 °C for 2 min. For Cas9 RNP cleavage and dA-tailing, dephosphorylated genomic DNA samples were treated with Cas9 RNPs, Taq polymerase (NEB, #M0273S), and dATP (NEB, #N0440S) at 37 °C for 30 min, followed by 72 °C for 5 min. For native barcode ligation, native barcoding expansion 1–12 (Oxford Nanopore Technologies, EXP-NBD104) were ligated to cleaved and dA-tailed genomic DNAs using Blunt/TA Ligase Master Mix (NEB, #M0367L) at room temperature for 10 min, followed by purification with Agencourt AMPure XP Beads (Beckman Coulter, #A63880) on a magnet. AMII adapters were ligated to barcoded genomic DNA using Quick T4 DNA ligase (NEB, #E7185A) at room temperature for 10 min, followed by purification with AMPure XP Beads on a magnet. The DNA library from Cas9-targeted native barcoding was primed into a MinION Flow Cell (FLO-MIN106D) on a MinION Mk1C and sequencing was performed for 20–21 h.

The crRNA design tool, CHOPCHOP [17], was used to design crRNAs, which were synthesized by Integrated DNA Technologies as follows: CR1, 5′gataccgacagcaatagtcc3′; CR2, 5′gtccttcagcactccacatc3′; CR3, 5′ctataggatccacagggagg3′; and CR4, 5′tgtcaaggtttggcttatag3′.

### Data analysis

Bases were called from Fast5 files using Guppy to generate Fastq files. Alignment to the reference sequence, which contains 10 D4Z4 RUs and flanking sequences from 3950 bp upstream of CR1 to 251 bp downstream of CR4, was conducted using Minimap2. Reference sequences were constructed using SnapGene software (from Insightful Science; available at snapgene.com). For DNA methylation analysis, sense- and antisense-strand reads from the 4qA and 10q loci were re-aligned to the corresponding reference sequences and then Nanopolish was performed [18]. Reference sequences contained the detected size of D4Z4 RUs and flanking sequences from 327 bp downstream of CR2 to 1 bp upstream of CR3. Unipro UGENE free software and Integrative genomics viewer were used for sequence alignment [19, 20]. For analysis of correlation between the distal D4Z4 CpG methylation rate and clinical symptoms, we calculated mean CpG methylation rates of the most distal D4Z4 RUs (RU3, RU2, and the promoter region of RU1) for all 4qA-reads obtained from each FSHD1 sample. Mean methylation rate or D4Z4 length, and age at disease onset or age at hospital inspection were analyzed and plotted with Graphpad Prism, and correlation coefficients were calculated by linear regression.

## Results

### Determination of numbers of D4Z4 RUs in patients with facioscapulohumeral muscular dystrophy by Nanopore sequencing

**Fig. 1:**
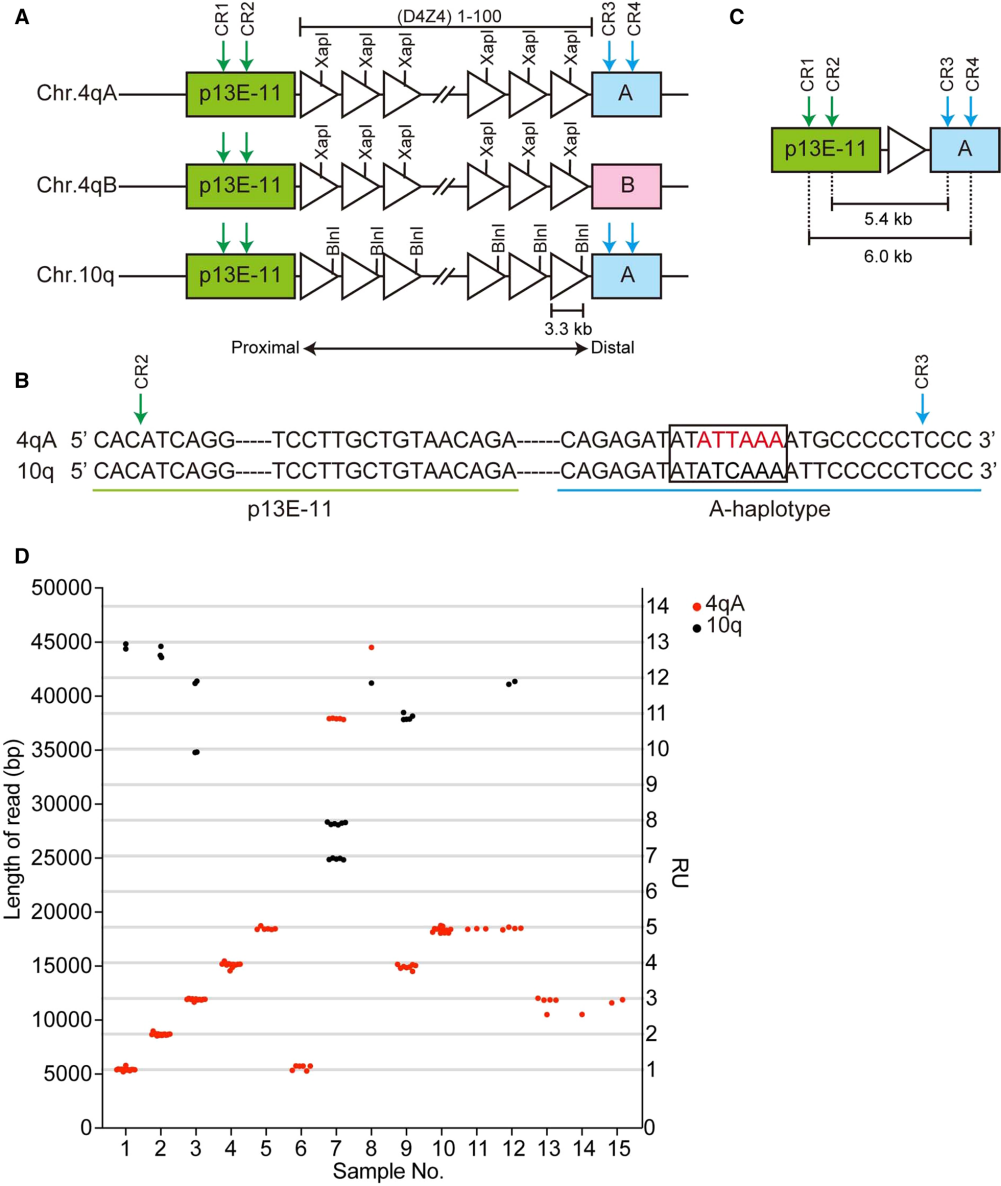
Determination of numbers of D4Z4 RUs in patients with facioscapulohumeral muscular dystrophy by Nanopore sequencing. **A** Schematic showing the D4Z4 repeat regions at the human chromosome 4qA, 4qB, and 10q loci. D4Z4 RUs are represented by triangles. The XapI and BlnI restriction enzyme sites are unique to chromosomes 4 and 10, respectively. The p13E-11, A-type haplotype, and B-type haplotype regions are indicated in green, blue, and pink, respectively. Green and blue arrows indicate crRNA cleavage sites (CR1/CR2/CR3/CR4). **B** The CR2 site in p13E-11 and CR3 site in the A-haplotype on the 4qA and 10q allele are shown by green and blue arrows, respectively. The 4qA polyadenylation signal is indicated in red. Sequences in the rectangle were used to distinguish reads from the 4qA and 10q loci. **C** Fragments carrying a single D4Z4 RU produced by CR2/CR3 or CR1/CR4 cleavage were 5.4 and 6.0 kb, respectively. **D** The length of identified reads and numbers of D4Z4 RUs are plotted. Red and black dots indicate reads derived from the 4qA and 10q loci, respectively

For CAS9 cleavage, we designed two types of guide RNA each for the p13E-11 (CR1/CR2) and A-haplotype (CR3/CR4) regions; the distal guides, CR3 and CR4, specifically recognized the 4qA and 10q loci, but not 4qB (Fig. 1A, B). To validate the nCATS assay, we analyzed five samples (Sample 1–5) from patients genetically diagnosed with FSHD1 by Southern blotting (Tables 1 and 2). Reads derived from the 4qA locus were obtained after alignment to the reference sequence, and the number of D4Z4 RUs were calculated from the read length (Fig. 1C; red dots, Fig. 1D; Additional file 1: Table S1). Sample 1, 2, 3, 4, and 5 carried 1, 2, 3, 4, and 5 D4Z4 RUs, respectively, consistent with results from Southern blotting.

**Table 1:** Patient clinical information

Sample ID	Patient ID	Genetic diagnosis	Age ranges at hospital inspection (years)	Sex	Age ranges at onset (years)	Asymmetric weakness	Facial weakness	Scapula weakness	Humeral weakness	Beevor’s sign	Other symptoms	Serum CK (IU/L)
1, 6	1	FSHD1	11–15	M	Birth	+	+	+	+	No data	Severe hearing loss	935
2	2	FSHD1	56–60	M	11–15	+	+	+	+	−	−	87
3	3	FSHD1	11–15	M	11–15	+	+	+	+	+	−	786
4	4	FSHD1	15–20	M	10–15	+	+	+	+	+	−	986
5	5	FSHD1	51–55	M	16–20	+	+	+	+	−	−	288
7	6	Suspected FSHD2	26–30	M	16–20	+	+	+	+	No data	−	1195
8	7	Suspected FSHD2	41–45	F	41–45	+	+	+	+	−	Mild hearing loss	380
9	8	Suspected FSHD1	16–20	M	11–15	+	+	+	+	−	−	887
10	9	Suspected FSHD1	61–65	F	41–45	+	+	+	+	+	−	259
11	10	Suspected FSHD1	71–75	F	46–50	+	+	+	+	−	Mild hearing loss	156
12	11	Suspected FSHD1	11–15	M	11–15	+	−	+	+	−	−	1262
13	12	Suspected FSHD1	21–25	F	Childhood	+	+	+	+	No data	−	241
14	13	Suspected FSHD1	16–20	F	Childhood	+	+	+	+	+	−	267
15	14	Suspected FSHD1	66–70	F	11–15	+	+	+	+	+	−	462

Genetic diagnosis was based on the results of Southern blotting (Table 2). Beevor’s sign indicates lower abdominal muscles weakness

*CK* creatine kinase

**Table 2:** Biospecimens and results of routine genetic analyses

Sample ID	Patient ID	Biospecimens for genomic DNA isolation	Southern blot (kb)				Bisulfite sequencing	Variant in *SMCHD1*
			RU	EcoRI with P13E-11 probe	EcoRI/BlnI with P13E-11 probe	HindIII with 4qA probe	Methylation rate (%)	
1	P1	PBL	1	10	7	17	54	Not analyzed
2	P2	PBL	2	13	10	20	Not analyzed	Not analyzed
3	P3	PBL	3	17	14	24	Not analyzed	Not analyzed
4	P4	PBL	4	20	17	27	Not analyzed	Not analyzed
5	P5	PBL	5	23	20	30	Not analyzed	Not analyzed
6	P1	Fibroblasts	–	Not analyzed	Not analyzed	Not analyzed	Not analyzed	Not analyzed
7	P6	PBL	–	Undetected	Undetected	Undetected	14.8	c.1040+1G>A (heterozygous)
8	P7	PBL	–	Undetected	Undetected	Undetected	9.0	c.3274_3276+1delAAAG (heterozygous)
9	P8	PBL	3 or 4?	17	17	Undetected	Not analyzed	Not analyzed
10	P9	PBL	2 or 5?	23	10	30	38.1	Not analyzed
11	P10	PBL	4 or 5?	20	20	40	Not analyzed	Not analyzed
12	P11	PBL	4 or 5?	20	20	Undetected	35.4%	Not analyzed
13	P12	PBL	2 or 6	13	10	34	Not analyzed	Not analyzed
14	P13	PBL	2 or 3?	13	13	32	44.0%	Not analyzed
15	P14	PBL	3?	17	13	24	Not analyzed	Not analyzed

*PBL* peripheral blood lymphocytes

In addition to the 4qA locus, we also occasionally obtained reads from chromosome 10q in Samples 1 (13 RUs), 2 (13 RUs), and 3 (10 RUs and 12 RUs) (black dots in Fig. 1D and Additional file 1: Table S2). We confirmed that both the 4qA- and 10q-derived reads were correctly assigned by identifying 4qA-specific (XapI, Non-BlnI, and pA) and 10q-specific (Non-XapI, BlnI, and Non-pA) sequences, along with the common p13E-11 sequence (Additional file 1: Fig. S1). Moreover, we confirmed that identical results were obtained using genomic DNA samples from the same subject from different sources by comparing Samples 1 and 6. These results suggest that our method enables precise determination of the number of D4Z4 RUs and the haplotypes on which the repeats reside.

We also analyzed samples that were undiagnosed by Southern blotting following linear gel electrophoresis because we failed to detect 4qA-derived bands (Samples 7 and 8) or failed to determine repeat lengths based on restriction fragment sizes (Samples 9–15) (Table 2). Using nCATS, we successfully determined the repeat lengths of 4qA-derived reads even from these challenging samples, as follows: Sample 7, 11 RUs; Sample 8, 13 RUs; Sample 9, 4 RUs; Sample 10, 5 RUs; Sample 11, 5 RUs; Sample 12, 5 RUs; Sample 13, 3 RUs; Sample 14, 3 RUs; and Sample 15, 3 RUs (Fig. 1D).

### A genomic deletion detected in patients with contracted D4Z4 repeats

**Fig. 2:**
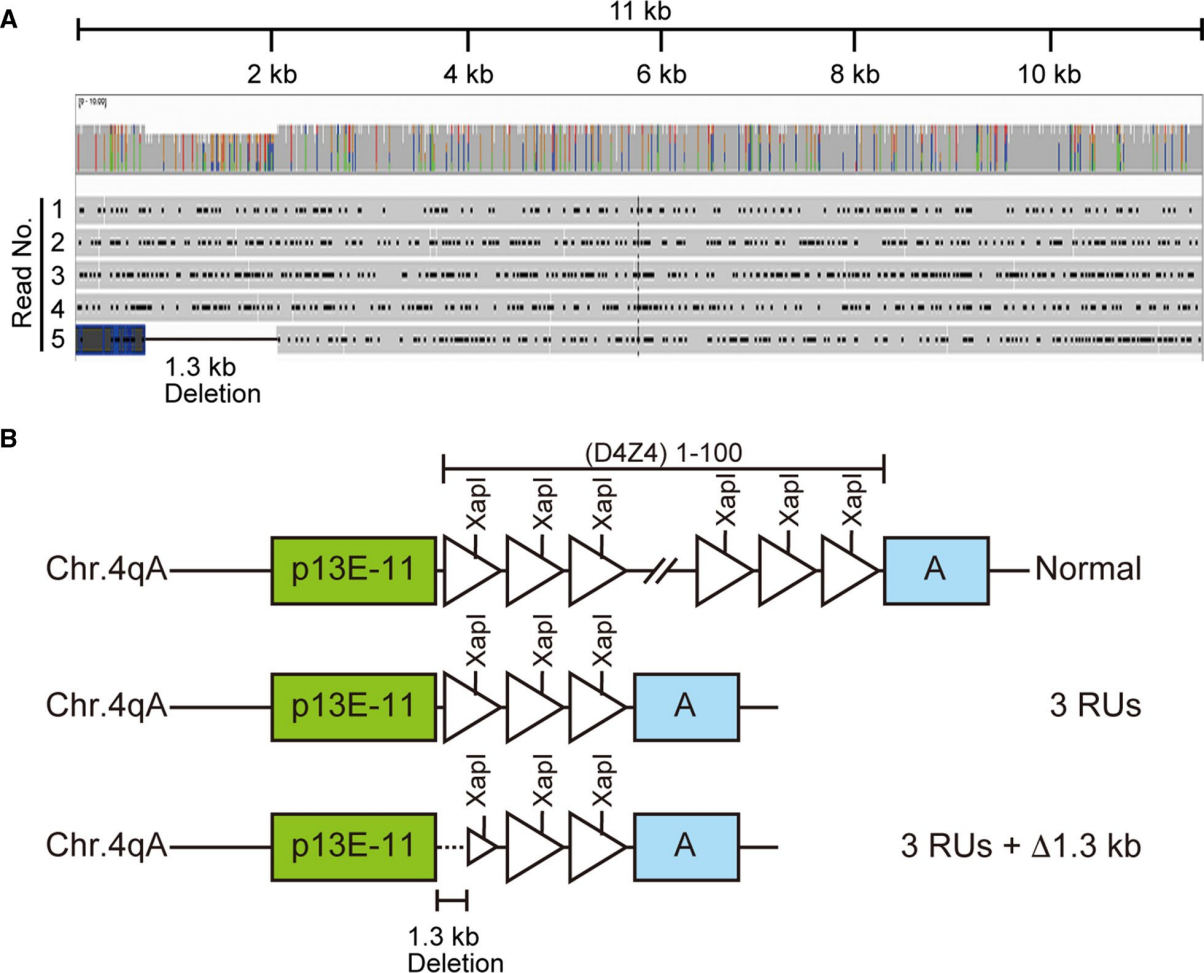
A genomic deletion detected in patients with contracted D4Z4 repeats. **A** Representative data showing reads obtained from Sample 13 mapped using Integrative Genomics Viewer. Among five reads (No. 1–5) with three repeat units (RUs) (11 kb), four reads (No. 1–4) with no deletion and one read (No. 5) with a 1.3 kb deletion were obtained. **B** The deletion was localized downstream of p13E-11 and extended to the middle of the most proximal D4Z4 RU

Interestingly, we also detected a genomic deletion, as an atypical cause of rearrangement of D4Z4 repeats. Samples 13 and 14 each generated one read with an intermediate size between 2 and 3 RUs. Sequence analysis revealed that both reads contained a deletion spanning 1.3 kb from 469 bases proximal to the most proximal D4Z4 RU to 859 bases within it (Fig. 2). Deletion within D4Z4 repeats has not been reported previously in FSHD1.

### CpG methylation rates in D4Z4 RUs

**Fig. 3:**
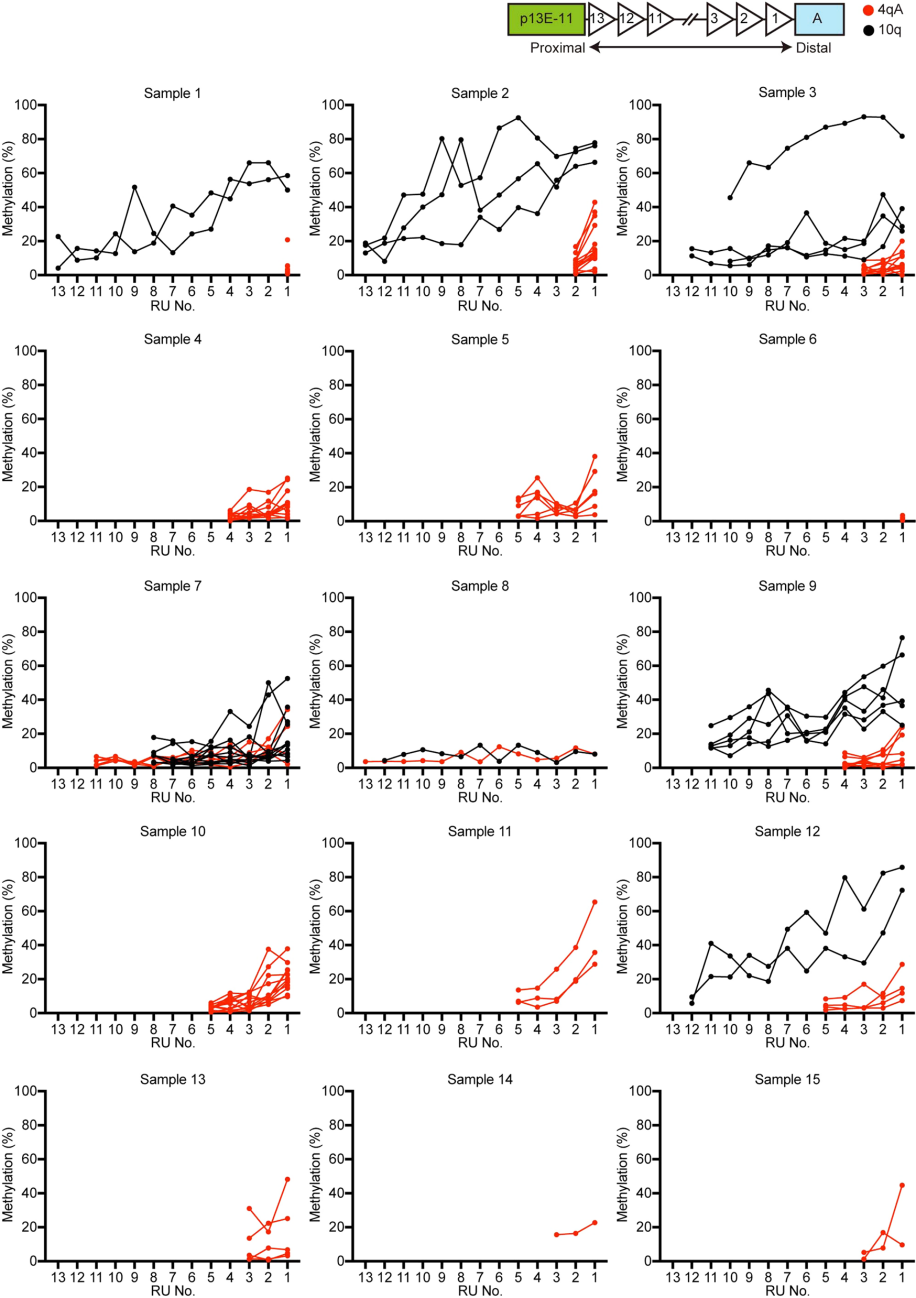
CpG methylation rates in D4Z4 RUs. CpG methylation rates of D4Z4 RUs in individual reads from the 4qA and 10q loci are plotted in red and black, respectively. D4Z4 RUs are numbered from the distal D4Z4 region

We also used nCATS results to determine the CpG methylation status of individual reads; therefore, we calculated CpG methylation rates for each RU in 4qA and 10q-derived reads (Fig. 3 and Additional file 1: Table S3). In FSHD1, the methylation rates of contracted 4qA-reads were consistently low, although those of the most distal D4Z4 RU at position 1 were relatively higher in most reads (Fig. 3). By contrast, the methylation rates of 10q-derived reads were low in proximal RUs, but elevated toward distal RUs. Further, in FSHD2, the CpG methylation rates of both 4qA- and 10q-reads were low throughout, with the exception of a few reads, in which the most distal RU1 was relatively highly methylated.

### Methylation rates in the promoter region and gene body of the most distal D4Z4 RU

**Fig. 4:**
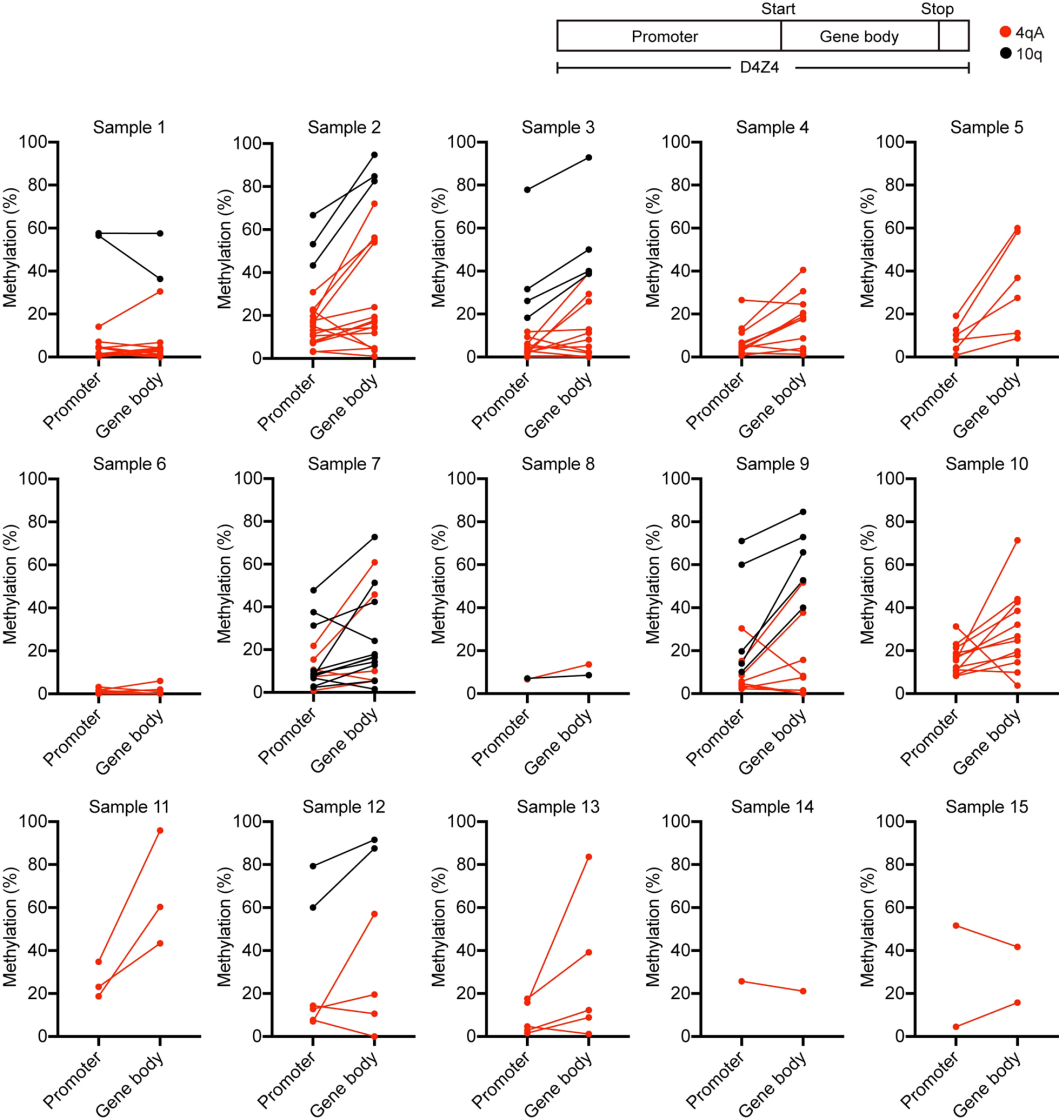
Methylation rates in the promoter region and gene body of the most distal D4Z4 RU. CpG methylation rates in the promoter and gene body of the most distal D4Z4 RU in individual reads are plotted. Reads from 4qA and 10q are shown in red and black, respectively

Next, we analyzed the CpG methylation rates of the promoter region and gene body of the most distal D4Z4 RU (RU1) separately (Fig. 4). Although Samples 1 and 6, which contained only one RU, showed similar CpG methylation rates in the promoter region and gene body, the methylation rates of promoter regions were generally lower than those in the gene body in all other samples from patients with both FSHD1 and FSHD2.

### Correlation between CpG methylation rate in distal D4Z4 and patient phenotypes

**Fig. 5:**
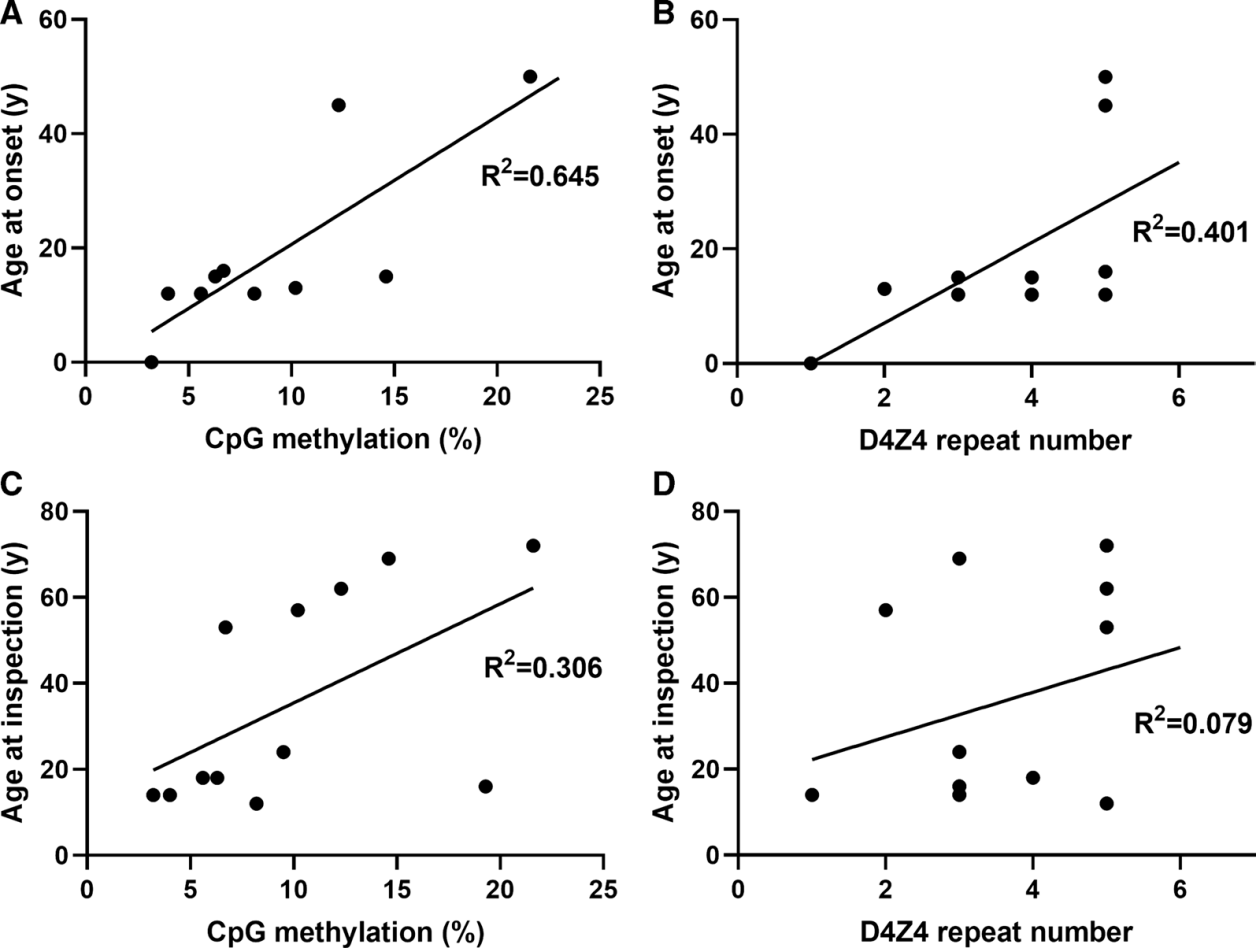
Correlation between CpG methylation rate in distal D4Z4 or repeat length, and patient phenotypes. **A**, **C** Scatter plots of mean CpG methylation rate in distal D4Z4 repeat units and age at disease onset (**A**) or age at hospital inspection (**C**) in FSHD1. **B**, **D** Scatter plots of D4Z4 repeat number and age at disease onset (**B**) or age at hospital inspection (**D**) in FSHD1. Correlation coefficients were calculated by linear regression. Two samples were removed due to unknown exact ages at onset of the disease

Epigenetic changes in the contracted D4Z4 repeats on chromosome 4qA have been observed previously and are considered to be associated with the development of FSHD1 [10, 13, 14]. We hypothesized that the CpG methylation rate of the most distal D4Z4 RUs is a determinant of disease development; therefore, we examined the correlation between average methylation rate of the most distal three RUs (Fig. 3) and patient age at onset or at hospital inspection. As shown in Fig. 5, we found a moderate correlation between CpG methylation and age at onset (R^2^ = 0.645) than that between D4Z4 repeat length and age at onset (R^2^ = 0.401). Although the correlation coefficient between CpG methylation and age at hospital inspection was not high (R^2^ = 0.306), there was a tendency toward correlation, in that CpG methylation rate < 10% was associated with hospital inspection at a younger age (≤ 20 years old), while CpG methylation rates of 10–20% were associated with that at > 40 years old.

## Discussion

In general, nCATS could be applicable to any other genetic disorders. In particular, it has an advantage on diagnosis of repeat-associated disorders, such as Huntington disease, spinal cerebellar ataxia, neuronal intranuclear inclusion disease, oculopharyngeal distal myopathy and others, in which the causative genetic variation cannot be identified by short read sequencing. In fact, it has been applied for analysis of some tandem repeat disorders, fragile X syndrome and myotonic dystrophy [21–23]. Nanopore sequencing was previously applied for analysis of FSHD using a bacterial artificial chromosome clone containing 13 D4Z4 repeat units [24].

In this study, we developed a direct sequencing system using nCATS to analyze clinical samples from patients with FSHD. Our method is more efficient and can collect more detailed information than conventional method. Conventional method for diagnosis of FSHD is carried out by multiple Southern blots for detection of the size of 4q-derived D4Z4 repeat and haplotyping 4q, and by bisulfite sequencing for measurement of the CpG methylation rate. In contrast, our method enables us to simultaneously identify the number and the methylation rate of D4Z4 repeat unit and the haplotype derived from 4qA. Our system has several advantages. First, long read sequencing can be applied to analysis of a similar DNA fragment size range to that detected by Southern blotting. Second, CRISPR/CAS9 enrichment allows barcoding sequencing of five samples simultaneously, saving time and cost. Third, single-molecule sequencing technology provides genetic information at the base level and can determine the number of RUs, even in samples that have mutated restriction enzyme sites, which prevent determination of RU number by the standard Southern blotting method. Finally, the nCATS system allows simultaneous detection of CpG methylation and D4Z4 RUs numbers, providing information about local epigenetic modification of D4Z4 repeats, due to the application of single-molecule sequencing of unamplified genomic DNA molecules derived from individual nuclei, without any bias.

Along with successful determination of D4Z4 RU numbers in patients, we also detected atypical rearrangement of D4Z4 repeats. As shown in Figs. 1D and 2, two reads of intermediate size had a 1.3 kb deletion in the most proximal D4Z4 RU, while p13E-11 was not deleted. This deletion is unlikely to be associated with the contraction of D4Z4 repeats in FSHD1, as the pathogenic alleles in FSHD1 usually maintain the intact RU structure, even when they contracted. Common atypical rearrangements found in individuals with FSHD1 have been reported, including D4Z4 proximally extended deletion (DPED1–7) alleles, which span 5.9–45.7 kb proximal to and within D4Z4, including p13E-11. In some DPED alleles, genetic elements, such as *DUX4C*, *FRG2*, *DBE-T*, and myogenic enhancers, are deleted, suggesting that their role in FSHD pathogenesis requires reevaluation [25].

The most important finding in our study was detection of DNA methylation rates across entire contracted and normal expanded D4Z4 repeat sequences from the 4qA and 10q loci. As shown in Fig. 3, 4qA-derived contracted reads were uniformly hypomethylated in patients with FSHD1, while both 4qA- and 10q-derived reads were uniformly hypomethylated in FSHD2, with the exception of a few reads. These results are similar to those generated in previous studies by Southern blot and bisulfite sequencing analyses [10, 13, 14], but our approach allows assessment of focal methylation rate at the nucleotide level. We further analyzed 10q-derived reads in FSHD1, and found that the methylation level was lower at proximal D4Z4 RUs (position 8–13), while it gradually increased (up to ≥ 60%) at distal RUs (positions 1–7). Given the mimicry of normal expanded 4qA-D4Z4 repeats by 10q-derived reads, these results suggest that only DNA hypermethylation at distal D4Z4 RUs contributes to suppression of the *DUX4* gene in the normal 4qA allele, while contraction of D4Z4 repeats causes hypomethylation of distal D4Z4 similar to proximal D4Z4 in the 10q locus, leading to DUX4 expression and consequent development of FSHD1. Indeed, mean CpG methylation rate of the most distal RUs and disease onset in patients was well-correlated. A larger study of the relationships among methylation rate, D4Z4 contraction, and clinical phenotypes is needed. To this end, we aim to overcome the limitation of decreased acquisition of sequencing reads from alleles with more than 10 RUs.

## Limitations

The nCATS method has limitations. First, the number of sequencing reads containing mildly contracted D4Z4 repeats (11–13 RUs) detected was quite low, particularly as only a few reads were obtained from the normal 10q locus, and no reads were obtained from some samples. The reasons why we could not obtain read from chromosome 10 in all samples and the number of reads in various samples are different are; (1) the difficulty to purify intact high molecular weight DNA, because the longer DNA might tend to be subject to degradation, (2) the difficulty to obtain longer DNA fragments beyond 13 RUs, because we used only the reads harboring full-length D4Z4 repeat in our analysis, (3) the efficacy of CAS9 cleavage of hypermethylated DNA, because distal D4Z4 were extremely higher methylation rates. Technical improvements in terms of preparation of genomic DNAs are required to overcome this shortcoming. Second, our method does not isolate reads derived from 4qB. Although the lack of analysis on 4qB is not likely to affect our conclusion, the epigenetic status in 4qB could be meaningful information as reference data for methylation rate of 4qA-derived D4Z4.

## Conclusions

In this study, we successfully determined the hypomethylation of D4Z4 RUs in individual 4qA fragments in FSHD. The hypomethylation in the contracted D4Z4 in FSHD1 provides a good explanation why the shortening of D4Z4 repeats is associated with severe phenotypes in patients and it induces abnormal DUX4 expression which leads to developing FSHD. For a further improvement, we need to have a large cohort of patients and controls in the future, which might give a clue for complete understanding of the pathomechanism of FSHD.

## Supplementary Information

**Additional file 1: Fig. S1.** Characteristic sequences detected by nCATS. Sequences of representative (A) 4qA- and (B) 10q-derived reads obtained from the indicated samples. The XapI/non-XapI and BlnI/non-BlnI sites in the most distal D4Z4 RU are shown. In Samples 8, 14, and 15, the XapI, XapI and non-BlnI, and non-XapI sites, respectively, in the second most distal D4Z4 RU are shown, due to the difficulty in identifying restriction sites. **Table S1.** Lengths of reads derived from the 4qA locus in each patient. **Table S2.** Lengths of reads derived from the 10q locus in each patient. **Table S3.** Methylation rates across all D4Z4 RUs at the 4qA and 10q loci.

## Data Availability

All data generated or analysed during this study are included in this published article and its Additional files.

## Abbreviations

FSHD: Facioscapulohumeral muscular dystrophy
RUs: Repeat units
nCATS: Nanopore CRISPR/Cas9-targeted resequencing
