# Supplementary material for: Simultaneous measurement of the size and methylation of chromosome 4qA-D4Z4 repeats in facioscapulohumeral muscular dystrophy by long-read sequencing
Source: J Transl Med. 2022 Nov 8;20:517. doi: 10.1186/s12967-022-03743-7 (PMC9644496; doi:10.1186/s12967-022-03743-7)
Supplement: Supplementary file 1 — Additional file 1: Fig. S1. Characteristic sequences detected by nCATS. Sequences of representative (A) 4qA- and (B) 10q-derived reads obtained from the indicated samples. The XapI/non-XapI and BlnI/non-BlnI sites in the most distal D4Z4 RU are shown. In Samples 8, 14, and 15, the XapI, XapI and non-BlnI, and non-XapI sites, respectively, in the second most distal D4Z4 RU are shown, due to the difficulty in identifying restriction sites. Table S1. Lengths of reads derived from the 4qA locus in each patient. Table S2. Lengths of reads derived from the 10q locus in each patient. Table S3. Methylation rates across all D4Z4 RUs at the 4qA and 10q loci. [file 12967_2022_3743_MOESM1_ESM.pdf]

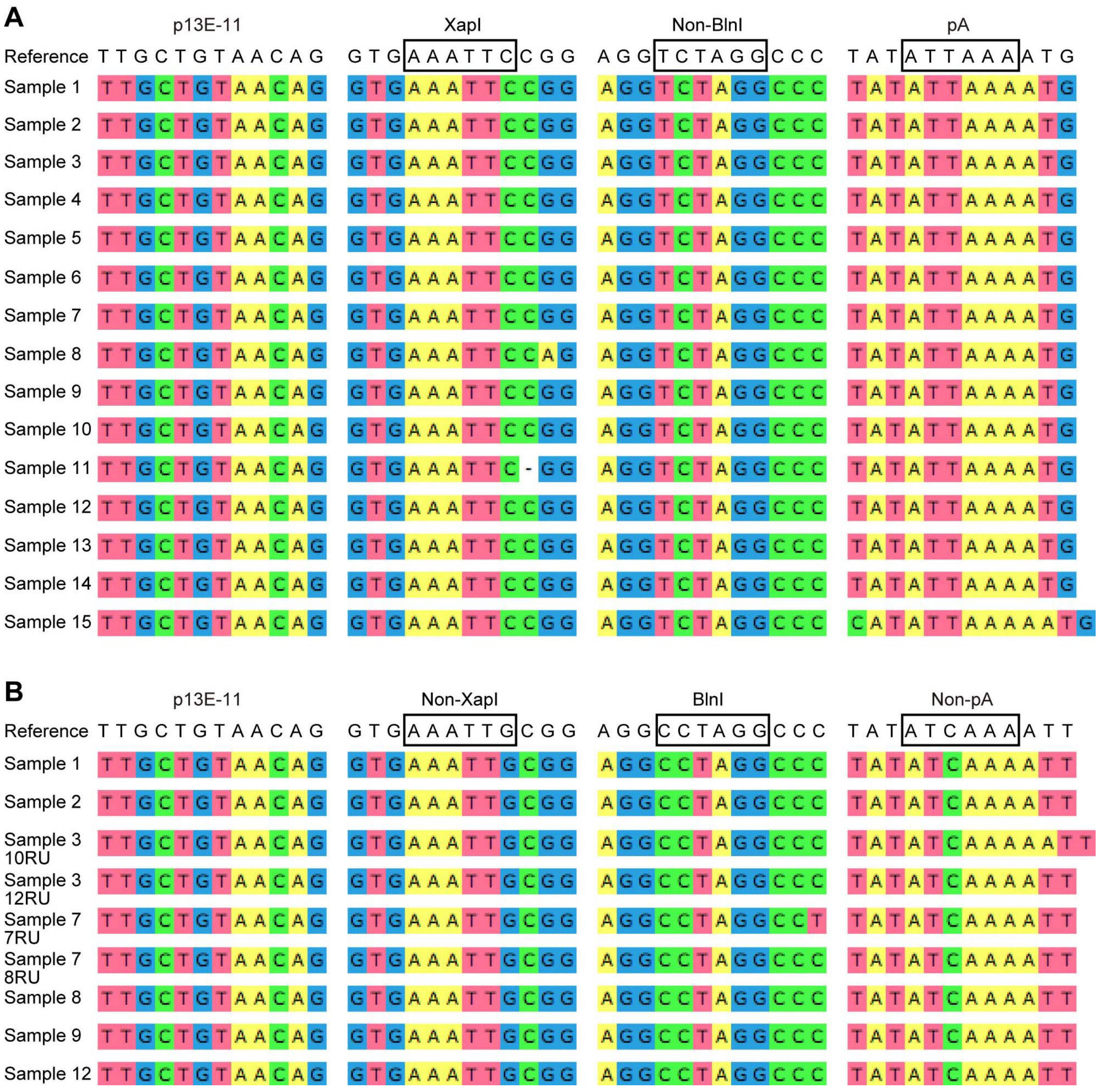

Fig. S1. Characteristic sequences detected on nCATS. (A) The sequences in the 4qA-derived representative read, and (B) the sequences in the 10q-derived representative read obtained from indicated samples. The XapI/non-XapI and BlnI/non-BlnI sites in the most distal D4Z4 RU are shown. In Samples 8, 14 and 15, the XapI site, the XapI and non-BlnI sites, and the non-XapI site in the second most distal D4Z4 RU, respectively are shown due to the difficulty to identify the restriction sites.

Table S1. The length of reads derived from 4qA locus in each patient.

Sample ID	Direction	Length (bp)
Sample 1	AntiSense	5221
	Sense	5316
	AntiSense	5372
	AntiSense	5390
	Sense	5391
	Sense	5397
	Sense	5425
	AntiSense	5428
	AntiSense	5434
	Sense	5440
	AntiSense	5449
	Sense	5469
	Sense	5794
Sample 2	Sense	8535
	AntiSense	8575
	AntiSense	8591
	AntiSense	8611
	Sense	8619
	AntiSense	8658
	AntiSense	8676
	AntiSense	8679
	Sense	8681
	AntiSense	8700
	Sense	8703
	Sense	8720
	AntiSense	8721
	Sense	8732
	AntiSense	8976
Sample 3	AntiSense	11659
	AntiSense	11861
	AntiSense	11872
	AntiSense	11912
	AntiSense	11922
	AntiSense	11923
	AntiSense	11924
	Sense	11952
	Sense	11967
	Sense	12010
	AntiSense	11909
Sample 4	AntiSense	14566
	Sense	14863
	Sense	15102
	AntiSense	15102
	AntiSense	15134
	Sense	15163
	AntiSense	15165
	AntiSense	15177

	AntiSense	15185
	AntiSense	15217
	AntiSense	15466
Sample 5	Sense	18383
	AntiSense	18397
	AntiSense	18404
	AntiSense	18448
	Sense	18453
	Sense	18730
Sample 6	AntiSense	5281
	Sense	5337
	AntiSense	5735
	Sense	5737
	Sense	5742
	AntiSense	5756
Sample 7	AntiSense	37844
	AntiSense	37903
	Sense	37923
	AntiSense	37926
	Sense	37959
Sample 8	Sense	44517
Sample 9	AntiSense	14511
	AntiSense	14792
	AntiSense	14851
	AntiSense	14903
	Sense	14951
	Sense	15044
	Sense	15133
	Sense	15157
Sample 10	Sense	18042
	Sense	18067
	AntiSense	18079
	AntiSense	18151
	Sense	18313
	AntiSense	18396
	Sense	18425
	AntiSense	18434
	AntiSense	18452
	AntiSense	18466
	AntiSense	18694
	Sense	18756
Sample 11	Sense	18450
	Sense	18460
	Sense	18412
Sample 12	Sense	18480
	AntiSense	18513
	AntiSense	18607
	Sense	18355
	Sense	12020

Sample 13	Sense	11873
	Sense	11838
	AntiSense	11838
	AntiSense	10505
Sample 14	AntiSense	10520
Sample 15	Sense	11596
	AntiSense	11878

Table S2. The length of reads derived from 10q locus in each patient.

Sample ID	Direction	Length (bp)
Sample 1	Sense	44383
	Antisense	44837
Sample 2	Antisense	43595
	Antisense	43780
	Sense	44612
Sample 3	Sense	34791
	Sense	34825
	Sense	41183
	Sense	41390
Sample 7	Sense	24845
	Sense	24872
	Antisense	24905
	Sense	24980
	Antisense	25002
	Sense	28081
	Antisense	28116
	Sense	28194
	Antisense	28246
	Sense	28301
	Antisense	28335
Sample 8	Sense	41213
Sample 9	Antisense	37844
	Antisense	37871
	Antisense	37879
	Sense	38166
	Sense	38487
Sample 12	Sense	41111
	Sense	41368

Table S3. The methylation rate at the entire D4Z4 RU of 4qA and 10q loci.

Sample ID	locus	Methylation rate (%)													
		D4Z4													
		13	12	11	10	9	8	7	6	5	4	3	2	1	
														Promoter	Gene body
Sample 1	4qA													0.9	4.4
														1.5	4.3
														1.6	0.0
														0.0	3.4
														4.4	0.0
														0.7	1.1
														0.0	3.7
														0.8	2.7
														14.1	30.5
														1.7	1.0
														4.2	6.8
														4.7	1.3
														7.1	4.2
	10q	22.7	8.8	10.1	24.4	13.8	18.9	40.6	35.3	48.4	44.9	66.0	66.1	56.5	36.4
		4.1	15.7	14.3	12.7	51.7	24.5	13.2	24.3	27.0	56.4	53.8	56.1	57.6	57.5
Sample 2	4qA												8.5	8.0	16.9
													6.4	7.1	14.7
													9.1	30.9	55.0
													1.7	10.5	11.9
													2.8	3.4	1.0
													5.6	22.1	3.9
													8.6	16.7	54.0
													4.5	11.4	19.4
													13.2	19.6	72.0
													2.8	17.6	23.9
													7.1	13.1	14.3
													0.8	3.1	4.8
													6.1	15.0	4.8
													16.8	22.8	56.3
													4.1	7.4	17.7
	10q	13.0	18.8	21.5	22.1	18.5	17.9	34.0	26.9	39.7	36.2	55.9	64.0	43.3	82.5
		17.5	21.8	47.1	47.6	80.3	52.8	57.3	86.5	92.5	80.6	69.8	72.5	66.7	84.8
		18.9	8.1	27.8	40.0	47.3	79.6	38.2	47.1	56.7	65.5	51.8	74.6	53.2	94.8
Sample 3	4qA											0.7	3.0	5.8	1.9
												2.8	3.7	6.1	39.1
												1.3	2.7	9.4	2.4
												5.6	0.4	2.8	0.0
												3.7	6.9	3.2	25.9
												3.5	1.2	1.7	29.4
												3.3	7.9	4.3	4.8
												1.3	4.4	0.0	0.0
												8.8	8.9	11.8	12.9
												0.6	1.3	2.4	8.1
												1.4	2.5	1.9	11.3
	10q				8.2	10.0	14.8	16.0	11.6	14.7	21.6	20.0	34.8	18.3	38.7
					45.5	66.0	63.4	74.6	81.0	87.0	89.3	93.1	92.9	77.9	92.9
			15.5	13.2	15.6	9.5	11.9	19.1	36.6	18.7	15.0	18.6	47.4	26.1	40.0
			11.3	6.8	5.5	6.1	17.2	16.3	10.7	12.5	11.2	9.1	16.8	31.6	50.0
											0.0	4.8	8.2	13.3	40.5
											6.1	18.5	16.9	26.5	24.5
											3.5	1.9	3.7	11.4	30.6
											0.4	7.8	1.6	0.8	4.1

Sample 4	4qA										2.8	2.6	4.7	5.6	2.7
											1.4	2.2	4.2	5.9	18.8
											4.7	4.0	3.7	2.8	20.5
											3.5	9.4	2.6	4.3	17.9
											4.1	4.8	11.7	4.3	8.7
											0.4	1.7	2.3	6.7	17.6
											3.8	2.0	3.9	1.9	1.3
Sample 5	4qA									3.1	1.7	4.5	2.8	0.9	8.7
										3.1	4.1	8.0	6.7	10.3	27.5
										2.9	15.9	9.9	4.0	8.0	11.2
										9.1	13.7	4.4	6.5	3.9	36.9
										13.7	17.0	5.6	10.7	12.6	58.4
										12.2	25.5	10.4	6.8	19.2	60.0
Sample 6	4qA													0.0	0.0
														1.7	0.0
														0.8	2.0
														0.9	0.0
														1.7	6.0
														3.2	1.0
Sample 7	4qA			6.6	4.4	2.4	6.9	6.1	8.6	6.2	13.7	4.4	17.1	21.8	60.9
				4.2	6.7	2.2	0.9	4.8	2.8	1.8	0.5	2.8	11.2	10.6	5.6
				4.1	5.0	2.3	7.0	3.8	3.5	4.5	6.4	15.2	12.2	7.4	10.0
				1.2	4.0	3.6	1.3	5.1	10.3	9.2	3.1	3.9	8.9	0.9	5.4
				1.7	5.1	1.3	6.4	1.9	6.3	6.0	2.6	8.9	11.6	15.4	45.8
	10q							2.8	1.4	2.1	4.8	3.0	6.8	2.8	12.8
								5.2	3.1	2.4	2.4	4.9	3.9	2.4	5.4
								3.8	2.6	3.3	3.7	1.3	8.4	6.7	1.5
								3.6	6.8	7.0	6.2	8.0	4.8	8.7	14.3
								1.5	5.1	3.4	4.9	4.6	5.3	7.5	51.3
							17.9	15.9	6.4	15.5	33.1	24.4	42.9	47.8	72.7
							3.4	2.0	2.3	11.6	12.2	6.9	5.3	8.0	16.4
							7.3	4.8	6.3	11.7	16.3	5.5	50.0	37.5	24.1
							2.8	3.5	2.9	7.2	9.0	6.0	7.2	7.6	16.7
							7.2	2.5	2.8	8.4	2.4	5.4	9.6	10.1	17.9
							9.0	14.3	15.4	12.4	11.6	18.2	8.5	31.3	42.4
Sample 8	4qA	3.6	3.8	3.7	4.2	3.6	9.1	3.5	12.4	8.2	4.8	5.6	11.7	6.7	13.6
	10q		4.3	7.9	10.6	8.3	6.5	13.3	3.8	13.3	9.0	3.1	9.5	7.1	8.6
Sample 9	4qA										8.8	6.1	10.6	30.4	8.3
											2.3	0.8	0.6	3.7	0.0
											1.2	1.3	2.0	15.2	51.7
											2.6	2.5	2.2	2.6	7.5
											0.0	4.0	8.5	8.6	37.7
											6.5	5.4	7.5	5.7	15.7
											0.5	4.8	0.0	4.7	0.0
											2.1	3.3	2.2	2.3	1.6
	10q			12.1	7.1	14.2	15.4	30.6	15.7	22.3	35.2	22.8	33.1	10.1	40.0
				24.8	29.5	35.9	43.6	20.1	20.9	22.8	41.8	47.7	41.1	71.0	84.6
				11.6	13.0	21.1	45.6	35.7	30.4	29.8	44.3	53.5	59.8	60.0	72.9
				12.8	16.2	17.7	12.6	16.1	20.0	20.9	40.0	33.3	46.0	19.7	52.8
				13.9	19.3	29.1	25.5	35.0	16.1	14.1	31.5	28.2	36.8	14.0	65.8
Sample 10	4qA									0.6	0.9	1.5	7.2	8.3	14.6
										3.7	7.1	12.4	37.6	21.4	38.5
										3.9	5.6	1.5	22.2	18.9	24.7
										4.0	2.5	6.5	9.9	17.4	26.7
										2.6	10.0	2.7	5.1	11.1	9.9
										4.0	1.8	2.7	9.3	16.5	32.1
										1.6	0.9	3.8	10.6	8.8	19.7

										0.8	2.4	10.5	7.3	31.3	3.8
										2.7	6.5	8.9	8.3	23.0	44.0
										2.7	8.4	4.0	6.9	12.3	17.9
										6.0	11.8	11.3	27.3	15.5	71.4
										4.9	8.8	12.4	17.3	10.4	42.6
Sample 11	4qA									6.4	8.9	8.2	18.8	23.1	43.4
										7.0	3.5	7.0	19.7	18.7	60.3
										13.6	14.7	25.9	38.6	34.8	95.9
Sample 12	4qA									4.6	4.8	3.2	11.6	7.0	57.0
										8.4	9.2	17.0	9.3	12.8	19.5
										3.7	2.5	3.2	6.0	14.4	10.6
										1.9	2.6	3.0	3.0	7.7	0.0
	10qA		5.8	41.0	33.6	22.0	18.7	49.4	59.3	47.0	79.7	61.2	82.4	79.3	91.5
			9.5	21.5	21.3	34.0	27.5	38.1	24.8	38.1	33.1	29.5	47.2	60.0	87.5
Sample 13	4qA											3.5	0.8	1.6	8.9
												1.2	7.8	2.9	12.3
												13.5	22.4	17.6	39.2
												31.0	17.3	15.8	83.6
												1.4	1.3	4.7	1.2
Sample 14	4qA											15.6	16.4	25.7	21.1
Sample 15	4qA											1.3	16.9	4.5	15.8
												5.2	7.8	51.6	41.7
